# The incidence of geriatric trauma is increasing and comparison of different scoring tools for the prediction of in-hospital mortality in geriatric trauma patients

**DOI:** 10.1186/s13017-020-00340-1

**Published:** 2020-10-19

**Authors:** Libing Jiang, Zhongjun Zheng, Mao Zhang

**Affiliations:** Department of Emergency Medicine, The Second Affiliated Hospital, Zhejiang University School of Medicine, Jiefang road 88, Hangzhou, China

**Keywords:** Elderly trauma, Geriatric trauma, Aging population, In-hospital mortality, Scoring tool

## Abstract

**Purpose:**

The study aimed to examine the changing incidence of geriatric trauma and evaluate the predictive ability of different scoring tools for in-hospital mortality in geriatric trauma patients.

**Methods:**

Annual reports released by the National Trauma Database (NTDB) in the USA from 2005 to 2015 and the Trauma Register DGU® in Germany from 1994 to 2012 were analyzed to examine the changing incidence of geriatric trauma. Secondary analysis of a single-center cohort study conducted among 311 severely injured geriatric trauma patients in a level I trauma center in Switzerland was completed. According to the in-hospital survival status, patients were divided into the survival and non-survival group. The differences of the ISS (injury severity score), NISS (new injury severity score), TRISS (Trauma and Injury Severity Score), APACHE II (Acute Physiology and Chronic Health Evaluation II), and SPAS II (simplified acute physiology score II) between two groups were evaluated. Then, the areas under the receiver-operating characteristic curve (AUC-ROC) of different scoring tools for the prediction of in-hospital mortality in geriatric trauma patients were calculated.

**Results:**

The analysis of the NTDB showed that the increase in the number of geriatric trauma ranged from 18 to 30% between 2005 and 2015. The analysis of the DGU® showed that the mean age of trauma patients rose from 39.11 in 1993 to 51.10 in 2013, and the proportion of patients aged ≥ 60 years rose from 16.5 to 37.5%. The findings from the secondary analysis showed that 164 (52.73%) patients died in the hospital. The ISS, NISS, APACHE II, and SAPS II in the death group were significantly higher than those in the survival group, and the TRISS in the death group was significantly lower than those in the survival group. The AUCs of the ISS, NISS, TRISS, APACHE II, and SAPS II for the prediction of in-hospital mortality in geriatric trauma patients were 0.807, 0.850, 0.828, 0.715, and 0.725, respectively.

**Conclusion:**

The total number of geriatric trauma is increasing as the population ages. The accuracy of ISS, NISS and TRISS was higher than the APACHE II and SAPS II for the prediction of in-hospital mortality in geriatric trauma patients.

## Background

Trauma is the fourth leading cause of death overall for all ages and the leading cause of death among young people aged less than 45 years [1]. Introduction of advanced trauma care system has significantly improved the outcome of trauma patients [2]. However, along with the aging population, geriatric trauma is rapidly becoming a major public health concern [3]. Geriatric trauma patients are more prone to poor prognosis due to complicated underlying diseases, various medication use, and limited physiological reserve [4–8]. A growing number of countries have pay attention to these populations, and some researchers even suggested it is necessary to build specific geriatric trauma centers [9–11]. However, there is lack of convincing evidence that the incidence of geriatric trauma is increasing. Additionally, accurate prognosis assessment is important for clinical decision-making in geriatric trauma patients [12–14]. A number of injury severity measures are developed for the purpose of outcome prediction [15], but few are validated in the geriatric trauma population. The study aimed to examine the changing incidence of geriatric trauma and evaluate the predictive ability of the ISS (injury severity score), NISS (new injury severity score), TRISS (Trauma and Injury Severity Score), APACHE II (Acute Physiology and Chronic Health Evaluation II) and SAPS II (simplified acute physiology score II) for the prediction of in-hospital mortality in geriatric trauma patients.

## Methods

The annual reports of the National Trauma Database (NTDB) in the USA from 2005 to 2015 (https://www.facs.org/quality-programs/trauma/tqp/center-programs/ntdb/docpub) and the Trauma Register DGU® in Germany from 1994 to 2012 (http://www.traumaregister-dgu.de/) were searched to examine the changing incidence of geriatric trauma. Through analysis of the NTDB, the proportion of geriatric trauma and corresponding death rates in the USA were calculated. Based on the data in the DGU® obtained by contacting the Prof. Dr. Rolf Lefering (Institut für Forschung in der Operativen Medizin; Fakultät für Gesundheit der Universität Witten/Herdecke), we calculated the mean age of trauma patients and the proportion of trauma patients aged ≥ 60 years and their trends over time. There is a lack of specific trauma database in China. Thus, we searched the annual reports of the National Bureau of Statistics of the People’s Republic of China (http://www.stats.gov.cn/), and the proportion of population aged ≥ 65 was calculated.

Secondary analysis of a single-center cohort study conducted among 311 severely injured geriatric trauma patients in a level I trauma center in Switzerland was completed [16, 17]. The authors in their original article evaluated the effects of standards of practice (SOP) on geriatric trauma patients [16, 17]. Categorical variables were represented as numbers and percentages, and continuous variables were represented as median (inter-quartile range). According to the in-hospital survival status, patients were divided into the survival and non-survival group. The differences of the ISS, NISS, TRISS, APACHE II, and SPAS II (Additional file 1 provides more details about these scoring tools) between two groups were evaluated. Then, the areas under the receiver-operating characteristic curve (AUC-ROC) of different scoring tools for the prediction of in-hospital mortality in geriatric trauma patients were calculated. Differences between groups at baseline were analyzed with the use of corresponding tests (two-sample *t* test or Mann-Whitney *U* test were used for continuous variables and chi-square test or Fisher’s exact test were used for categorical variables). All statistical process was performed using IBM SPSS 20.0 (Zhejiang University) and *P* < 0.05 was regarded as statistical significance.

## Results

### The incidence of geriatric trauma is increasing

**Fig. 1:**
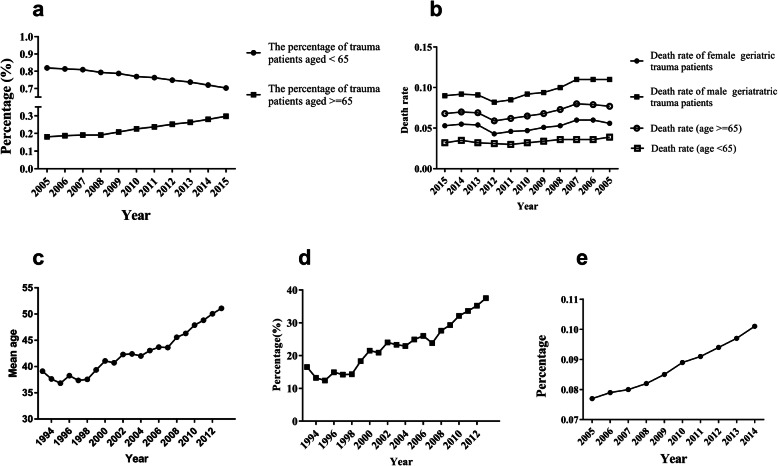
**a** The change trend of trauma patients aged < 65 vs ≥ 65 using the data of NTDB. **b** The mortality change trend of trauma patients aged < 65 vs ≥ 65 using the data of NTDB. **c**, **d** The age change trend of trauma patients using data of DGU. **e** The age change trend of Chinese people.

The analysis of the NTDB showed that the increase in the number of geriatric trauma ranged from 18 to 30% between 2005 and 2015. Meanwhile, the proportion of trauma patients aged less than 65 years declined during the same period (Fig. 1a). The death rate of geriatric trauma patients was significantly higher than that of their younger counterparts. Additionally, the results showed that men had a significantly higher mortality rate than women in geriatric trauma patients (Fig. 1b). Eighty nine thousand patients with ISS (injury severity score) ≥ 9 in the DGU® was analyzed. The mean age rose from 39.11 years in 1993 to 51.10 years in 2013, and the proportion of those aged ≥ 60 years rose from 16.5 to 37.5% between 1993 and 2013 (Fig. 1c, d). In China, major trauma accounts for more than 60 million visits annually to hospitals, and is responsible for 700,000 to 800,000 deaths per year [18]. Based on the statistics of National Bureau of Statistics of the People’s Republic of China (http://www.stats.gov.cn/), the proportion of the population who aged 65 or above increased from 7.7% to 10.1% between 2005 and 2014 (Fig. 1e).

### Comparison of different scoring tools for the prediction of in-hospital mortality in geriatric trauma patients

The sample included 311 patients aged ≥ 65 years [16, 17], while 59.00% were male. One hundred and sixty-four (52.73%) patients died in the hospital. Table 1 shows the detailed characteristics of included patients. There was no significant difference between the survival and death group, in terms of trauma mechanism, base excess, body mass index, leucocytes, thrombocytes, prothrombin, systolic blood pressure, mean artery pressure, and temperature. Patients in the death group were older than those in the survival group. The survivors of geriatric trauma had higher GCS (Glasgow coma Scale) and hemoglobin level, and lower lactate level than the patients who died. We found the length of ICU stay and length of hospital stay were significantly shorter in the death group compared to the survival group.

**Table 1:** Characteristics of included patients

	Survival	Death	***P*** value
Number of patient	147	164	
Trauma mechanism			
Blunt	144(48.30%)	154(51.70%)	0.07
Penetrating	3(23.10%)	10(76.90%)	
GCS			
3-8	47(27.30%)	125(72.7-%)	< 0.01
9-12	22(61.10%)	14(38.90%)	
13-15	78(75.70%)	25(24.30%)	
Age	74.00 (68.25 to 80.00)	78.00(72.00 to 83.00)	< 0.01
Base excess [mEq/L]	− 2.80 (− 5.50 to − 0.80)	− 3.25(− 7.80 to − 0.55)	0.32
Body mass index	25.95 (22.89 to 28.40)	25.90(22.04 to 29.16)	0.74
Hemoglobin [g/L]	11.80 (9.90 to 13.10)	10.60(8.10 to 12.20)	< 0.01
Lactate [mmol/L]	1.90 (1.15 to 2.80)	2.10(1.40 to 3.40)	0.03
Los of hospital [days]	16.50 (9.00 to 25.00)	1.50(1.00 to 3.00)	< 0.01
Leucocytes [10^9/L]	10.49 (7.33 to 13.96)	11.12(6.96 to 14.24)	0.86
Thrombocytes [10^9/L]	190.50(152.00 to 239.50)	174.00(132.00 to 230.00)	0.07
Prothrombin [% normal]	80.00 (59.00 to 95.00)	74.00(52.00 to 89.25)	0.08
Systolic pressure mmHg]	142.50 (115.00 to 160.000)	125.00(110.00 to 158.75)	0.09
Mean artery pressure [mmHg]	100.00 (82.00 to 112.75)	92.00(76.50 to 113.50)	0.22
Temperature	35.40(34.50 to 36.20)	35.25(34.10 to 36.00)	0.31
Los of ICU [days]	5.00 (2.00 to 12.00)	1.00(0.50 to 2.00)	< 0.01
Los of MV [days]	1.00(0.00 to 6.25)	1.00(0.25 to 2.00)	0.04

*GCS* Glasgow score, *Los* length of stay, *ICU* intensive care unit, *MV* mechanical ventilation

**Fig. 2:**
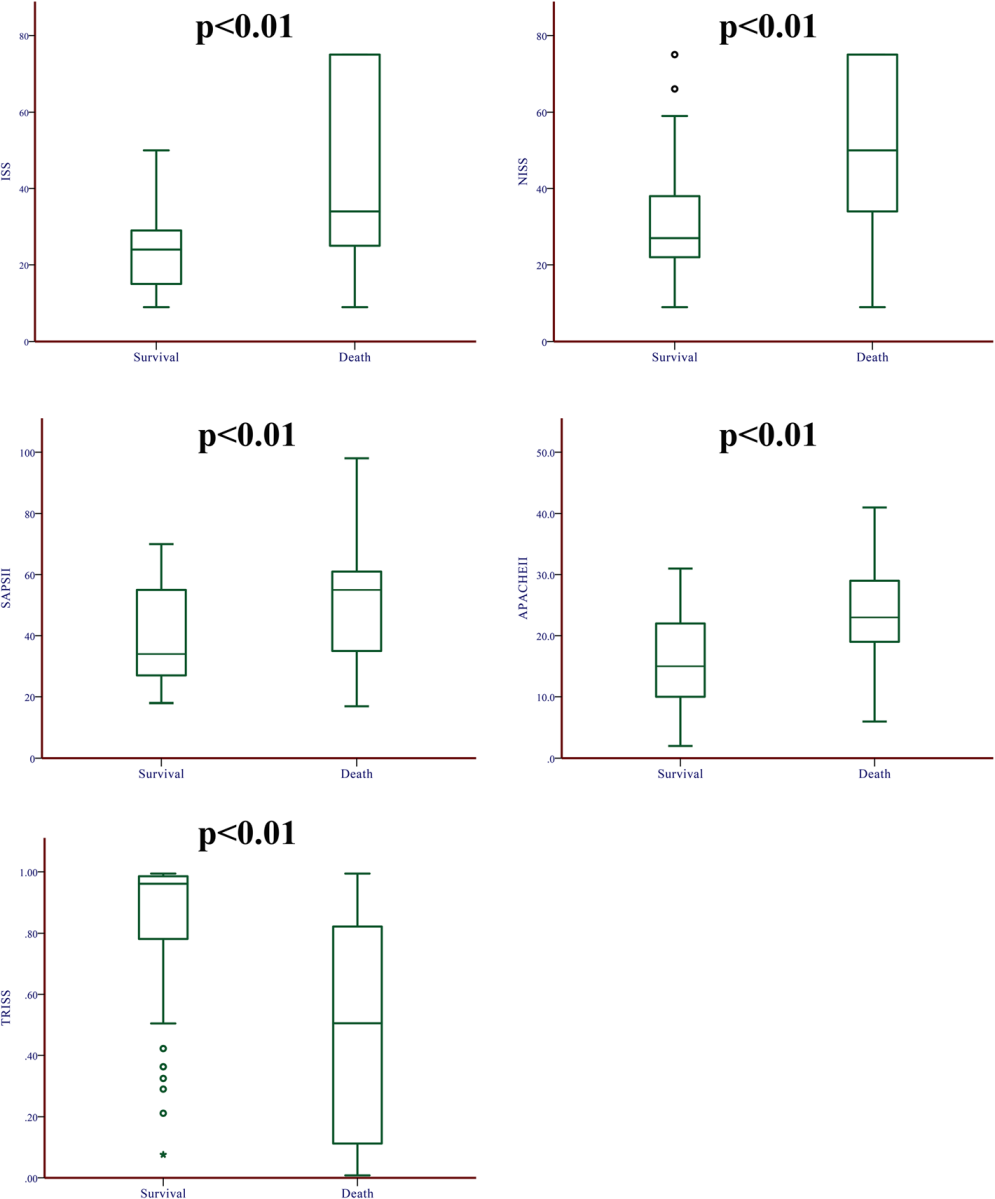
The comparison of ISS, NISS, APACHE II, SPAS II, and TRISS between the survival group and death group. ISS, injury severity score; NISS, new injury severity score; SPAS II, simplified acute physiology score II; APACHE II, Acute Physiology and Chronic Health Evaluation II; TRISS, Trauma and Injury Severity Score

The ISS (34.00 vs 24.00, *P* < 0.01), NISS (50.00 vs 27.00, *P* < 0.01), APACHE II (23.00 vs 15.00, *P* < 0.01), and SAPS II (55.00 vs 34.00, *P* < 0.01) in the death group were significantly higher than that in the survival group. The median TRISS was significantly lower in the death group than that in the survival group (0.51 vs 0.96, *P* < 0.01) (Table 2 and Fig. 2).

**Table 2:** Comparison of different scoring tools between two groups

	Survival	Death	***P*** value
**ISS**	24.00 (14.50 to 29.00)	34.00(25.00 to 75.00)	< 0.01
**NISS**	27.00 (22.00 to 38.00)	50.00(34.00 to 75.00)	< 0.01
**TRISS**	0.96(0.78 to 0.99)	0.51(0.11 to 0.82)	< 0.01
**APACHE II**	15.00 (10.00 to 22.00)	23.00(19.00 to 29.00)	< 0.01
**SPAS II**	34.00 (27.00 to 55.00)	55.00(34.75 to 61.00)	< 0.01
*SPAS II* simplified acute physiology score II, *APACHE II* Acute Physiology and Chronic Health Evaluation II, *ISS* injury severity score, *NISS* new injury severity score, *TRISS* Trauma and Injury Severity Score

**Fig. 3:**
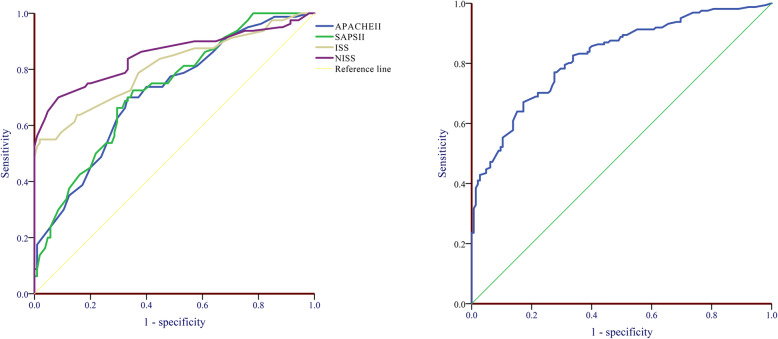
The AUC of ISS, NISS, APACHE II, SPAS II, and TRISS in predicting in-hospital mortality among geriatric trauma patients. ISS, injury severity score; NISS, new injury severity score; SPAS II, simplified acute physiology score II; APACHE II, Acute Physiology and Chronic Health Evaluation II; TRISS, Trauma and Injury Severity Score

The AUCs were calculated to assess the performance of different scoring tools for the prediction of in-hospital mortality in geriatric trauma patients. Table 3 and Fig. 3 shows the AUC of the ISS was 0.807, NISS was 0.850, TRISS was 0.828, APACHE II was 0.715, and SPAS II was 0.725. Table 4 shows the difference between the AUCs of different scoring tools. Compared with APACHE II and SAPS II, the ISS, NISS, and TRISS appear to be better predictors of in-hospital mortality in elderly trauma patients. Especially the AUCs of NISS and TRISS were significantly higher than that of the APACHE II and SPAS II (*P* < 0.01).

**Table 3:** Diagnostic value of different scoring tool in predicting in-hospital mortality

	AUC	95% CI of AUC
APACHE II	0.715	0.644 to 0.778
ISS	0.807	0.743 to 0.861
NISS	0.850	0.790 to 0.898
SPAS II	0.725	0.655 to 0.788
TRISS	0.828	0.766 to 0.880

*SPAS II* simplified acute physiology score II, *APACHE II* Acute Physiology and Chronic Health Evaluation II, *ISS* injury severity score, *NISS* new injury severity score, *TRISS* Trauma and Injury Severity Score, *AUC* area under the receiver operating characteristic curve

**Table 4:** The matrix of AUC comparison between different scoring tool using *P* value

	SPAS II	APACHE II	ISS	NISS	TRISS
SPAS II		0.61	0.07	< 0.01	< 0.01
APACHE II	0.61		0.03	< 0.01	< 0.01
ISS	0.07	0.03		0.02	0.34
NISS	< 0.01	<0.01	0.02		0.37
TRISS	< 0.01	< 0.01	0.34	0.37	

*SPAS II* simplified acute physiology score II, *APACHE II* Acute Physiology and Chronic Health Evaluation II, *ISS* injury severity score, *NISS* new injury severity score, *TRISS* Trauma and Injury Severity Score, *AUC* area under the receiver operating characteristic curve

## Discussion

With the increasingly aging of the population, the incidence of geriatric trauma tends to increase. The study aimed to examine the changing incidence of geriatric trauma and evaluate the predictive ability of different scoring tools for the prediction of in-hospital mortality in geriatric trauma patients. Firstly, through analysis of the annual reports of two large trauma databases, we found the mean age of trauma patients and the proportion of geriatric trauma patients are increasing. Then, secondary analysis of a cohort study showed that the ISS, NISS, and TRISS were better than the SAPS II and APACHE II for the prediction of in-hospital mortality in geriatric trauma patients.

Increased life expectancy and independent and active lifestyle expose a great number of elderly people to serious accidents [19, 20]. Additionally, the elderly may be more susceptible to injury due to multiple comorbidities and physical changes associated with aging (decreased vision, hearing loss, and some level of neurocognitive decline etc.). Kehoe et al. in their study also found the mean age of major trauma patients had increased from 36.1 years in 1990 to 53.8 years in 2013 [21]. It is predicted that nearly 40% of all injured patients are expected to exceed age 65 years by 2050 [22]. However, this prediction had been exceeded at Lehigh Valley Health Network in 2013 [23]. Bortz el al reported in their study that 46.6% of all trauma patients aged 65 or greater, and 17.7% aged 85 or greater [23]. Christopher et al. evaluated the effects of a new system on mortality in trauma patients in England, and they found the median age of the patients increased from 45 to 59 years between 2008 and 2017. Additionally, they found the proportion of major trauma patients aged 65 years or more increased from 22 to 42% during the study period [24]. Beck et al. described temporal trends in the incidence of major trauma in older adults, and they found that the proportion of patients with major trauma who were aged 65 years or more increased from 25·1 to 36·7% between 2007 and 2016 [25]. The incidence of geriatric trauma increased by 4.3% per year [25].

Accurate prognostic expectations are important for decision-making in geriatric trauma patients. Several scoring systems have been developed in an attempt to accurately predict outcomes for trauma patients [13]. The ISS is a widely recognized anatomical scoring system to assess injury severity, which is equal to the squared values of the three most severely injured body areas [26]. However, the ISS ignores the serious injuries that occurring in the same anatomical area. The NISS has been reported to be better than the ISS in measuring the severity of multiple trauma patients, which considers the three most serious injuries, regardless of body region [27–29]. The TRISS is the most commonly used score for benchmarking trauma fatality outcome, based on age, revised trauma score (RTS), and the ISS [30]. However, the RTS is difficult to be obtained in sedated/intubated patients. Most studies showed that the NISS and TRISS were better than the ISS for the prediction of outcome in trauma patients [31]. Yousefzadeh-Chabok et al. reported the AUCs of the ISS and TRISS in predicting mortality among geriatric trauma patients were 0.76 and 0.94 [32]. Homanna Javali et al. also compared the ISS, NISS, and TRISS for predicting mortality in cases of geriatric trauma, and the corresponding AUCs were 0.963, 0.970, and 0.972, respectively [31].

The APACHE II and SAPS II are generally used to measure the severity and predict outcome for critically ill patients. There was no significant difference between the APACHE II versus the TRISS for the prediction of mortality in severe trauma patients [33–36]. The results of the study by Reiter et al. showed the AUCs of the SAPS II and TRISS in predicting mortality in severe trauma patients were 0.87 and 0.84 [37]. Philipp et al. reported the AUCs of the TRISS and SAPS II for the prediction mortality in multiple-trauma patients was 0.83 and 0.86 [38]. Both studies found the combination of different scores might improve the predictive ability. However, there is no study comparing the performance differences between the APACHE II versus SAPS II in predicting death among geriatric trauma patients. Our study tested the performance of the ISS, NISS, TRISS, APACHE II, and SAPS II for the prediction of in-hospital mortality in geriatric trauma patients. The results suggested that the ISS, NISS, and TRISS might be superior to the APACHE II and SAPS II.

Some limitations of this study should be acknowledged. Firstly, only the annual reports of two large trauma databases were analyzed to describe the age profile of trauma patients, which may limit the generalization of the results. Then, the geriatric specific scoring system, known as the Geriatric Trauma Outcome Score (GTOS), was not evaluated in the present study [39, 40]. However, it has been reported that there was no significant performance difference between the GTOS and other general injury severity measures [12, 13]. Finally, it has the inherent limitations of a secondary analysis, so conclusions should be considered only as hypothesis-generating.

## Conclusion

The number and proportion of geriatric trauma patients are increasing rapidly. The ISS, NISS, and TRISS have better performance for the prediction of in-hospital mortality in geriatric trauma patients in comparison with the APACHE II and SAPS II.

## Supplementary information

**Additional file 1.** List of different scoring tool.

## Data Availability

The datasets used and analyzed during the current study are available from the corresponding author on reasonable request.

## Abbreviations

GCS: Glasgow score
Los: Length of stay
ICU: Intensive care unit
MV: Mechanical ventilation
SPAS II: Simplified acute physiology score II
APACHE II: Acute Physiology and Chronic Health Evaluation II
ISS: Injury severity score
NISS: New injury severity score
TRISS: Trauma and Injury Severity Score
AUC: Area under the receiver operating characteristic curve
RTS: Revised trauma score
NTDB: National trauma databank
ASCOT: A Severity Characterization of Trauma
MTOS: Major Trauma Outcome Study
GTOS: Geriatric Trauma Outcome Score
