# Supplementary material for: The incidence of geriatric trauma is increasing and comparison of different scoring tools for the prediction of in-hospital mortality in geriatric trauma patients
Source: World J Emerg Surg. 2020 Oct 19;15:59. doi: 10.1186/s13017-020-00340-1 (PMC7574576; doi:10.1186/s13017-020-00340-1)
Supplement: Supplementary file 1 — Additional file 1. List of different scoring tool. [file 13017_2020_340_MOESM1_ESM.doc]

**Additional file 1: List of different scoring tool**

Contents

[APACHE Ⅱ: 1](#__RefHeading___Toc50109992)

[SAPS Ⅱ: 1](#__RefHeading___Toc50109993)

[ISS: 1](#__RefHeading___Toc50109994)

[NISS: 1](#__RefHeading___Toc50109995)

[TRISS: 1](#__RefHeading___Toc50109996)

APACHE Ⅱ: https://www.mdcalc.com/apache-ii-score

SAPS Ⅱ: https://www.mdcalc.com/simplified-acute-physiology-score-saps-ii

ISS: https://www.mdcalc.com/injury-severity-score-iss

NISS: Injury severity of each of 6 body systems are scored according the Abbreviated Injury Scale (AIS). The NISS is simply the sum of squares of the three most severe injuries, regardless of body region injured.

TRISS: https://www.mdapp.co/trauma-injury-severity-score-triss-calculator-277/
